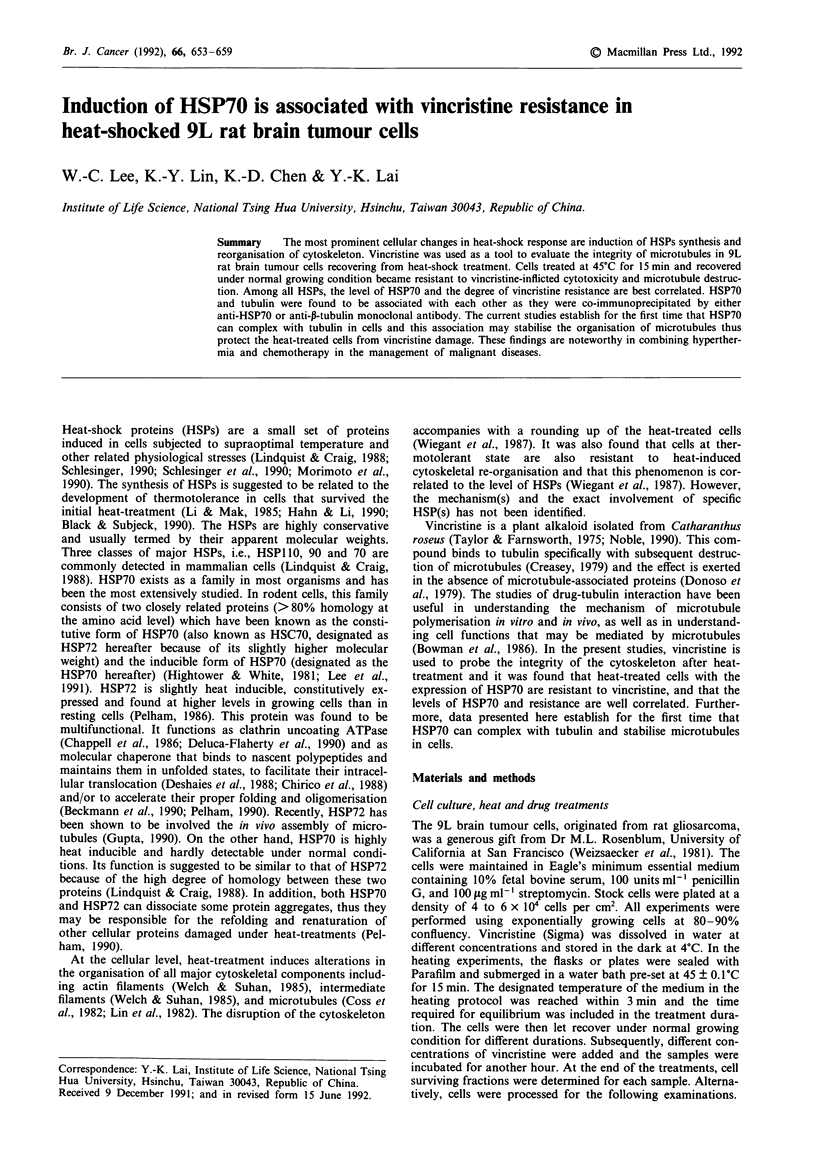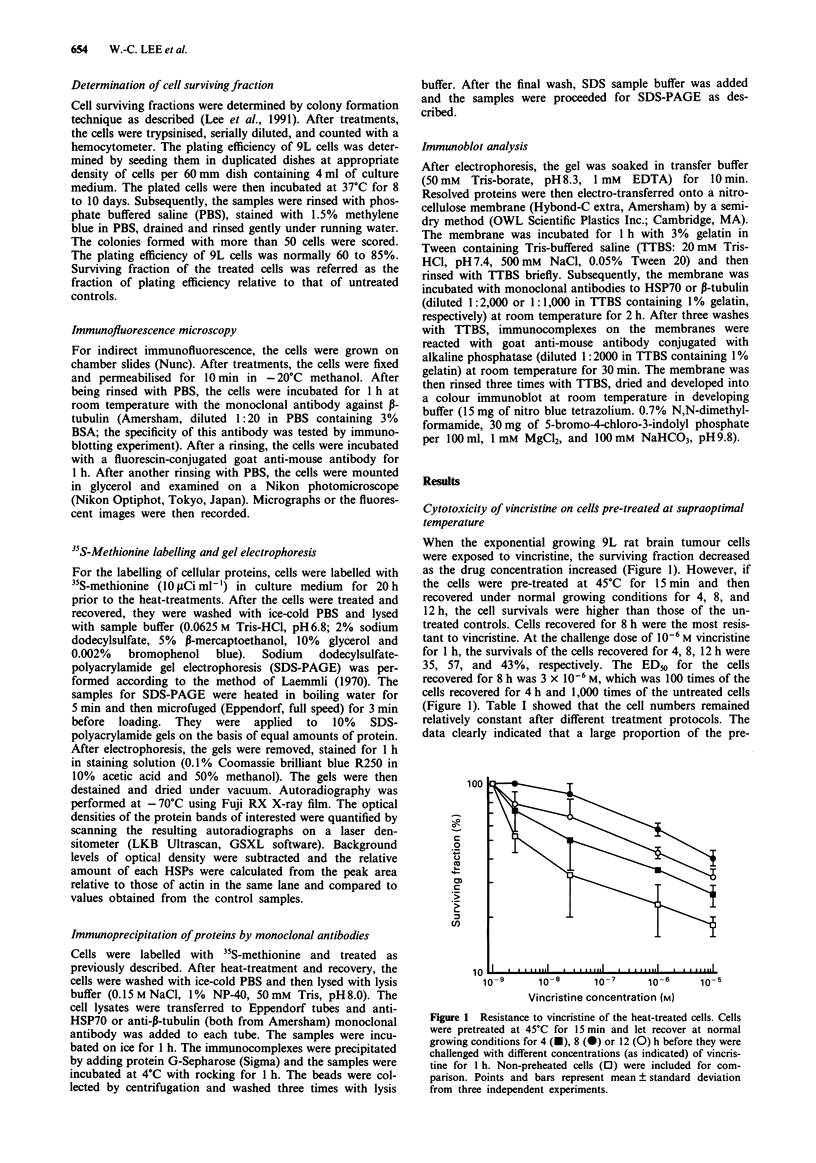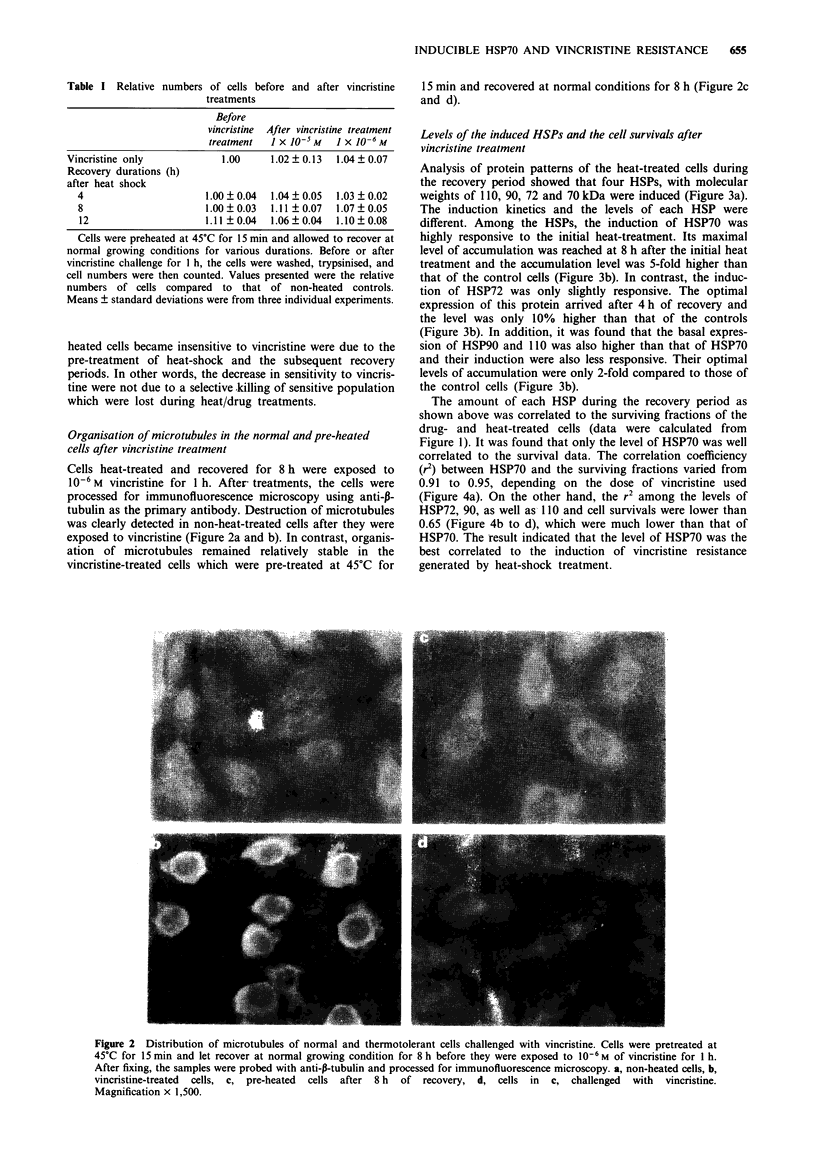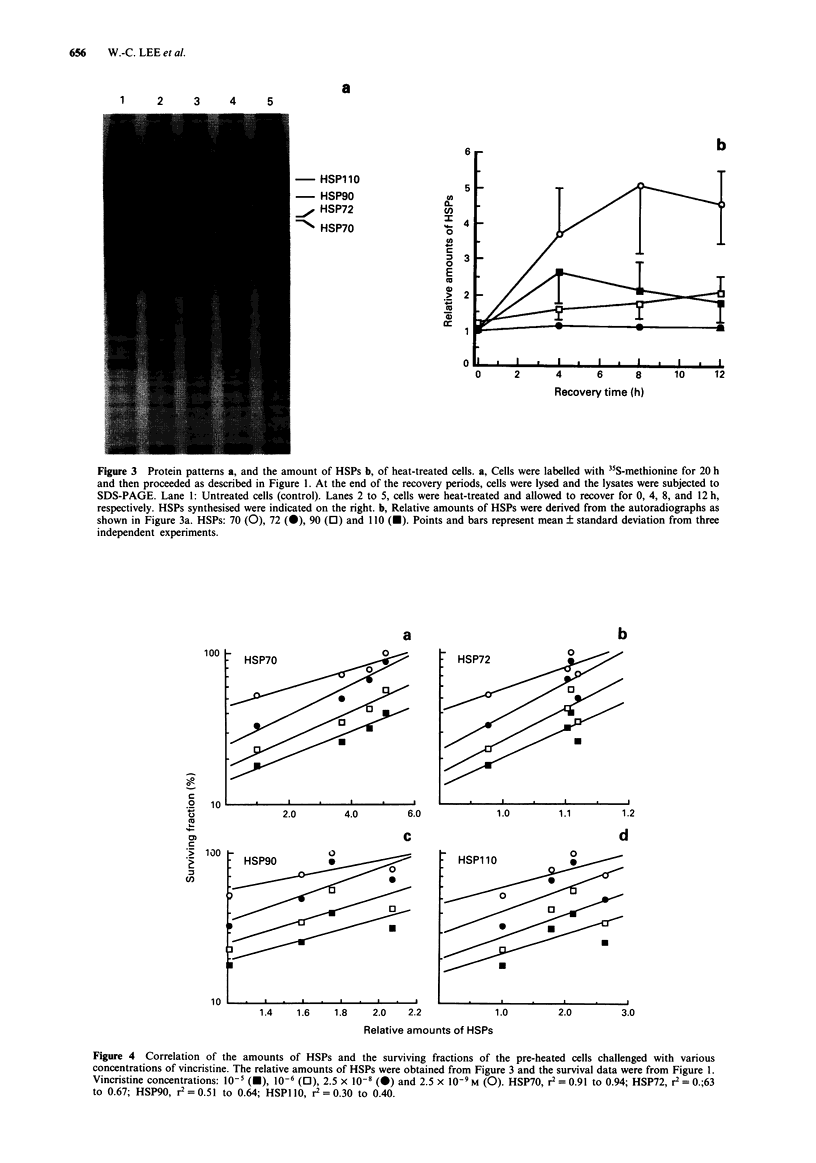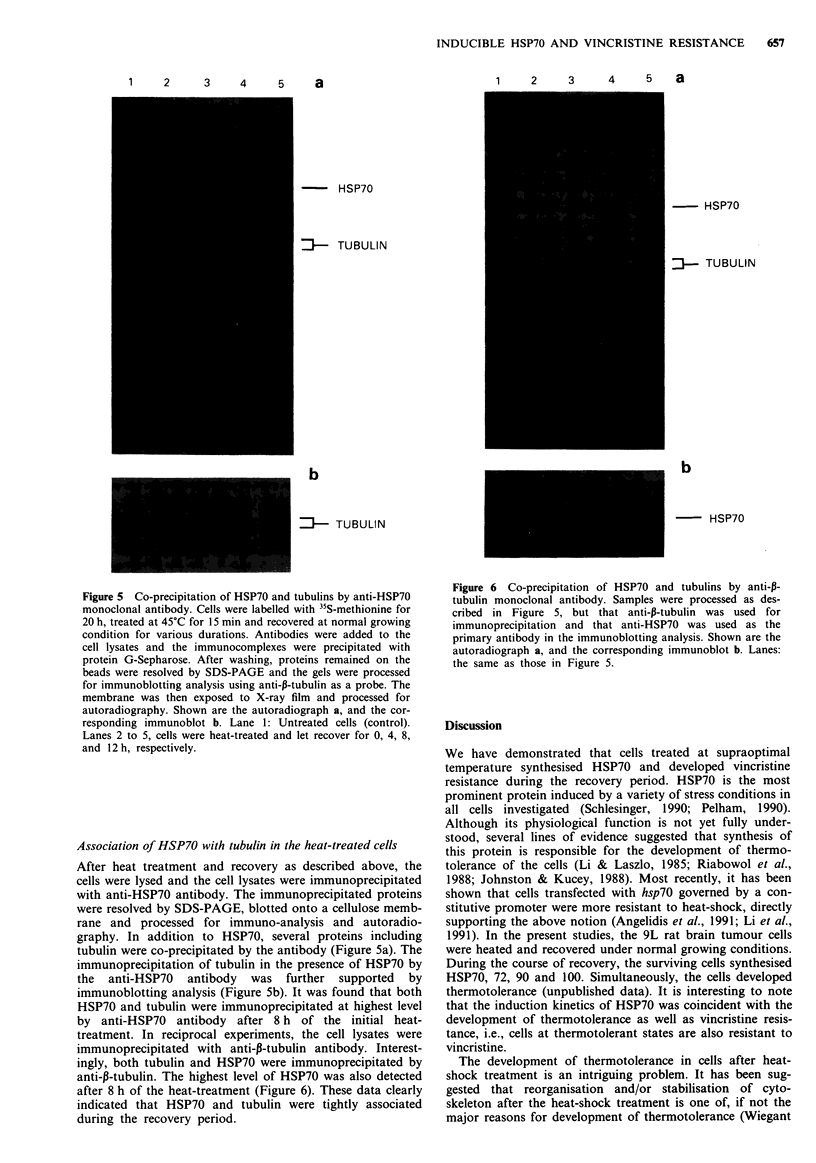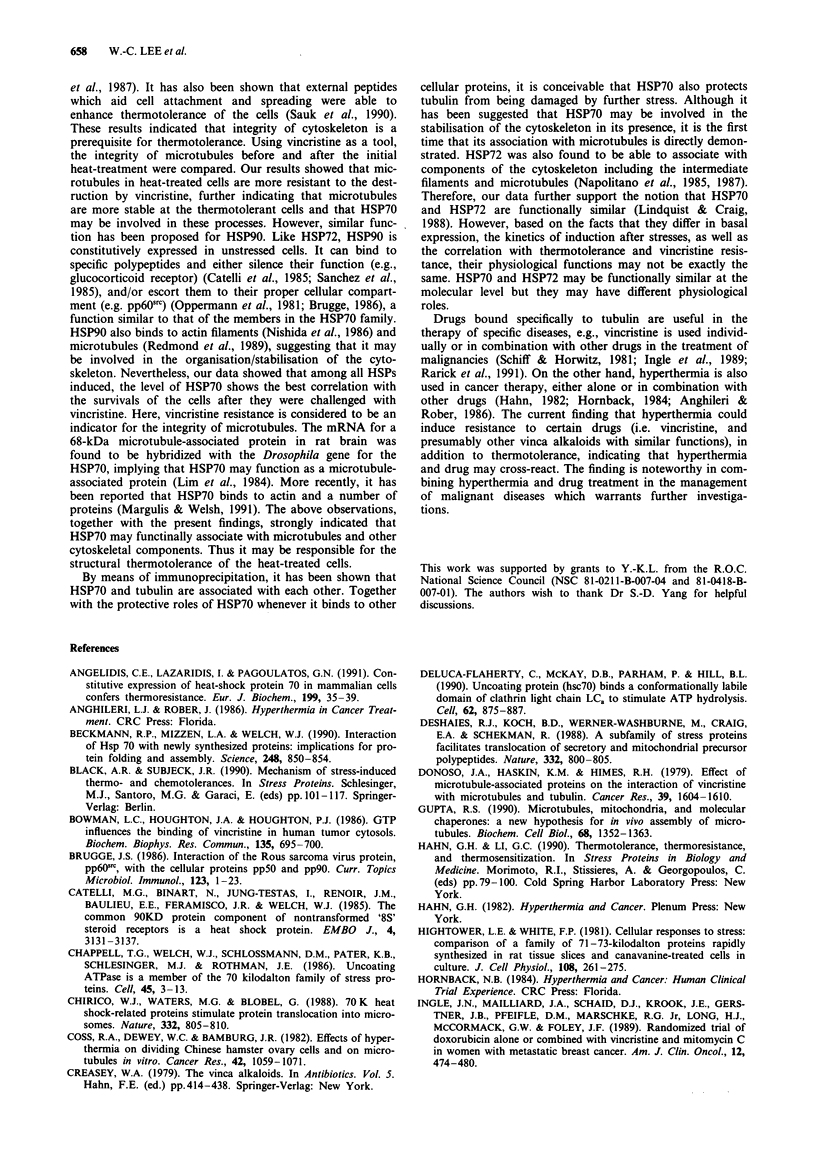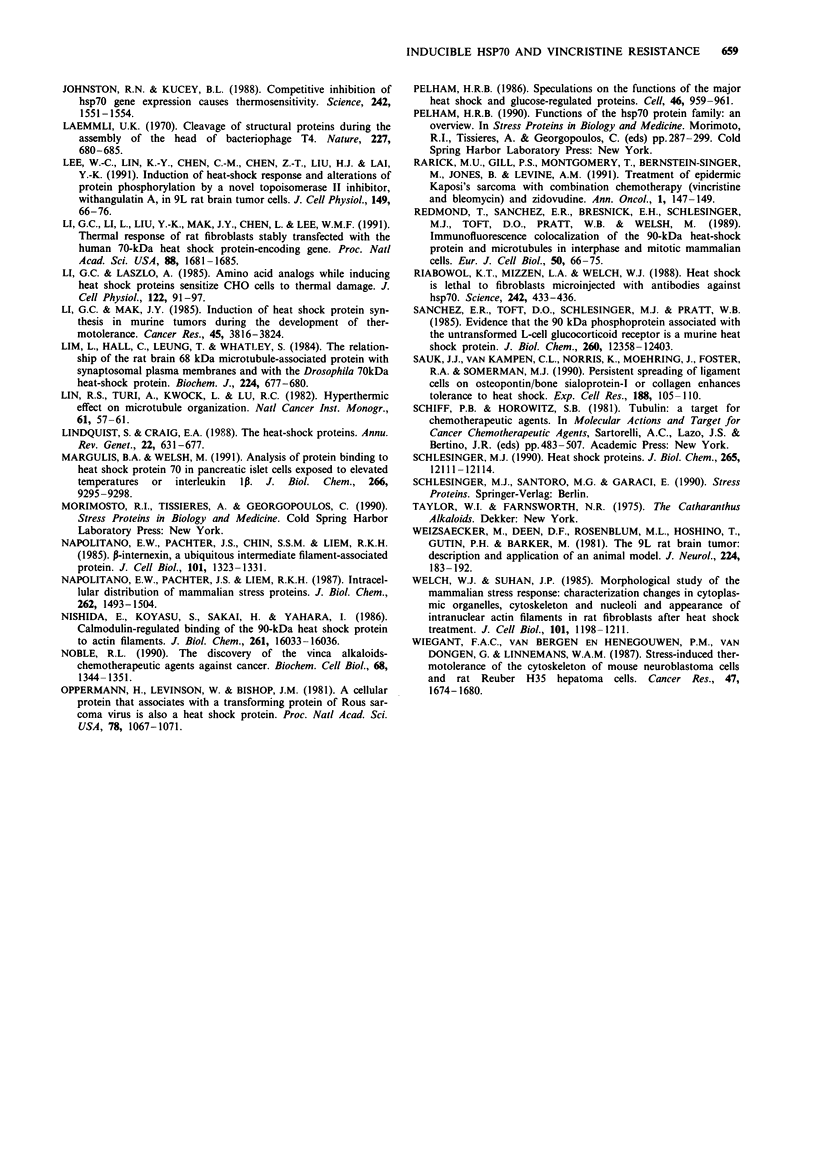# Induction of HSP70 is associated with vincristine resistance in heat-shocked 9L rat brain tumour cells.

**DOI:** 10.1038/bjc.1992.332

**Published:** 1992-10

**Authors:** W. C. Lee, K. Y. Lin, K. D. Chen, Y. K. Lai

**Affiliations:** Institute of Life Science, National Tsing Hua University, Hsinchu, Taiwan, Republic of China.

## Abstract

**Images:**


*Br. J. Cancer* (1992), **66**, 653–659 © Macmillan Press Ltd., 1992

### Induction of HSP70 is associated with vincristine resistance in heat-shocked 9L rat brain tumour cells

W.-C. Lee, K.-Y. Lin, K.-D. Chen & Y.-K. Lai

*Institute of Life Science, National Tsing Hua University, Hsinchu, Taiwan 30043, Republic of China.*

**Summary** The most prominent cellular changes in heat-shock response are induction of HSPs synthesis and reorganisation of cytoskeleton. Vincristine was used as a tool to evaluate the integrity of microtubules in 9L rat brain tumour cells recovering from heat-shock treatment. Cells treated at 45°C for 15 min and recovered under normal growing condition became resistant to vincristine-inflicted cytotoxicity and microtubule destruction. Among all HSPs, the level of HSP70 and the degree of vincristine resistance are best correlated. HSP70 and tubulin were found to be associated with each other as they were co-immunoprecipitated by either anti-HSP70 or anti-β-tubulin monoclonal antibody. The current studies establish for the first time that HSP70 can complex with tubulin in cells and this association may stabilise the organisation of microtubules thus protect the heat-treated cells from vincristine damage. These findings are noteworthy in combining hyperthermia and chemotherapy in the management of malignant diseases.

Heat-shock proteins (HSPs) are a small set of proteins induced in cells subjected to supraoptimal temperature and other related physiological stresses (Lindquist & Craig, 1988; Schlesinger, 1990; Schlesinger *et al.*, 1990; Morimoto *et al.*, 1990). The synthesis of HSPs is suggested to be related to the development of thermotolerance in cells that survived the initial heat-treatment (Li & Mak, 1985; Hahn & Li, 1990; Black & Subjeck, 1990). The HSPs are highly conservative and usually termed by their apparent molecular weights. Three classes of major HSPs, i.e., HSP110, 90 and 70 are commonly detected in mammalian cells (Lindquist & Craig, 1988). HSP70 exists as a family in most organisms and has been the most extensively studied. In rodent cells, this family consists of two closely related proteins (> 80% homology at the amino acid level) which have been known as the constitutive form of HSP70 (also known as HSC70, designated as HSP72 hereafter because of its slightly higher molecular weight) and the inducible form of HSP70 (designated as the HSP70 hereafter) (Hightower & White, 1981; Lee *et al.*, 1991). HSP72 is slightly heat inducible, constitutively expressed and found at higher levels in growing cells than in resting cells (Pelham, 1986). This protein was found to be multifunctional. It functions as clathrin uncoating ATPase (Chappell *et al.*, 1986; Deluca-Flaherty *et al.*, 1990) and as molecular chaperone that binds to nascent polypeptides and maintains them in unfolded states, to facilitate their intracellular translocation (Deshaies *et al.*, 1988; Chirico *et al.*, 1988) and/or to accelerate their proper folding and oligomerisation (Beckmann *et al.*, 1990; Pelham, 1990). Recently, HSP72 has been shown to be involved the *in vivo* assembly of microtubules (Gupta, 1990). On the other hand, HSP70 is highly heat inducible and hardly detectable under normal conditions. Its function is suggested to be similar to that of HSP72 because of the high degree of homology between these two proteins (Lindquist & Craig, 1988). In addition, both HSP70 and HSP72 can dissociate some protein aggregates, thus they may be responsible for the refolding and renaturation of other cellular proteins damaged under heat-treatments (Pelham, 1990).

At the cellular level, heat-treatment induces alterations in the organisation of all major cytoskeletal components including actin filaments (Welch & Suhan, 1985), intermediate filaments (Welch & Suhan, 1985), and microtubules (Coss *et al.*, 1982; Lin *et al.*, 1982). The disruption of the cytoskeleton accompanies with a rounding up of the heat-treated cells (Wiegant *et al.*, 1987). It was also found that cells at thermotolerant state are also resistant to heat-induced cytoskeletal re-organisation and that this phenomenon is correlated to the level of HSPs (Wiegant *et al.*, 1987). However, the mechanism(s) and the exact involvement of specific HSP(s) has not been identified.

Vincristine is a plant alkaloid isolated from *Catharanthus roseus* (Taylor & Farnsworth, 1975; Noble, 1990). This compound binds to tubulin specifically with subsequent destruction of microtubules (Creasey, 1979) and the effect is exerted in the absence of microtubule-associated proteins (Donoso *et al.*, 1979). The studies of drug-tubulin interaction have been useful in understanding the mechanism of microtubule polymerisation *in vitro* and *in vivo*, as well as in understanding cell functions that may be mediated by microtubules (Bowman *et al.*, 1986). In the present studies, vincristine is used to probe the integrity of the cytoskeleton after heat-treatment and it was found that heat-treated cells with the expression of HSP70 are resistant to vincristine, and that the levels of HSP70 and resistance are well correlated. Furthermore, data presented here establish for the first time that HSP70 can complex with tubulin and stabilise microtubules in cells.

### Materials and methods

### *Cell culture, heat and drug treatments*

The 9L brain tumour cells, originated from rat gliosarcoma, was a generous gift from Dr M.L. Rosenblum, University of California at San Francisco (Weizsaecker *et al.*, 1981). The cells were maintained in Eagle's minimum essential medium containing 10% fetal bovine serum, 100 units $ml^{-1}$ penicillin G, and 100 $\mu g\ ml^{-1}$ streptomycin. Stock cells were plated at a density of 4 to 6 $\times 10^4$ cells per $cm^2$. All experiments were performed using exponentially growing cells at 80–90% confluency. Vincristine (Sigma) was dissolved in water at different concentrations and stored in the dark at 4°C. In the heating experiments, the flasks or plates were sealed with Parafilm and submerged in a water bath pre-set at 45 ± 0.1°C for 15 min. The designated temperature of the medium in the heating protocol was reached within 3 min and the time required for equilibrium was included in the treatment duration. The cells were then let recover under normal growing condition for different durations. Subsequently, different concentrations of vincristine were added and the samples were incubated for another hour. At the end of the treatments, cell surviving fractions were determined for each sample. Alternatively, cells were processed for the following examinations.

Correspondence: Y.-K. Lai, Institute of Life Science, National Tsing Hua University, Hsinchu, Taiwan 30043, Republic of China.
Received 9 December 1991; and in revised form 15 June 1992.

*Determination of cell surviving fraction*

Cell surviving fractions were determined by colony formation technique as described (Lee *et al.*, 1991). After treatments, the cells were trypsinised, serially diluted, and counted with a hemocytometer. The plating efficiency of 9L cells was determined by seeding them in duplicated dishes at appropriate density of cells per 60 mm dish containing 4 ml of culture medium. The plated cells were then incubated at 37°C for 8 to 10 days. Subsequently, the samples were rinsed with phosphate buffered saline (PBS), stained with 1.5% methylene blue in PBS, drained and rinsed gently under running water. The colonies formed with more than 50 cells were scored. The plating efficiency of 9L cells was normally 60 to 85%. Surviving fraction of the treated cells was referred as the fraction of plating efficiency relative to that of untreated controls.

*Immunofluorescence microscopy*

For indirect immunofluorescence, the cells were grown on chamber slides (Nunc). After treatments, the cells were fixed and permeabilised for 10 min in −20°C methanol. After being rinsed with PBS, the cells were incubated for 1 h at room temperature with the monoclonal antibody against β-tubulin (Amersham, diluted 1:20 in PBS containing 3% BSA; the specificity of this antibody was tested by immunoblotting experiment). After a rinsing, the cells were incubated with a fluorescin-conjugated goat anti-mouse antibody for 1 h. After another rinsing with PBS, the cells were mounted in glycerol and examined on a Nikon photomicroscope (Nikon Optiphot, Tokyo, Japan). Micrographs or the fluorescent images were then recorded.

*$^{35}$S-Methionine labelling and gel electrophoresis*

For the labelling of cellular proteins, cells were labelled with $^{35}$S-methionine (10 μCi ml$^{-1}$) in culture medium for 20 h prior to the heat-treatments. After the cells were treated and recovered, they were washed with ice-cold PBS and lysed with sample buffer (0.0625 M Tris-HCl, pH 6.8; 2% sodium dodecylsulfate, 5% β-mercaptoethanol, 10% glycerol and 0.002% bromophenol blue). Sodium dodecylsulfate-polyacrylamide gel electrophoresis (SDS-PAGE) was performed according to the method of Laemmli (1970). The samples for SDS-PAGE were heated in boiling water for 5 min and then microfuged (Eppendorf, full speed) for 3 min before loading. They were applied to 10% SDS-polyacrylamide gels on the basis of equal amounts of protein. After electrophoresis, the gels were removed, stained for 1 h in staining solution (0.1% Coomassie brilliant blue R250 in 10% acetic acid and 50% methanol). The gels were then destained and dried under vacuum. Autoradiography was performed at −70°C using Fuji RX X-ray film. The optical densities of the protein bands of interested were quantified by scanning the resulting autoradiographs on a laser densitometer (LKB Ultrascan, GSXL software). Background levels of optical density were subtracted and the relative amount of each HSPs were calculated from the peak area relative to those of actin in the same lane and compared to values obtained from the control samples.

*Immunoprecipitation of proteins by monoclonal antibodies*

Cells were labelled with $^{35}$S-methionine and treated as previously described. After heat-treatment and recovery, the cells were washed with ice-cold PBS and then lysed with lysis buffer (0.15 M NaCl, 1% NP-40, 50 mM Tris, pH 8.0). The cell lysates were transferred to Eppendorf tubes and anti-HSP70 or anti-β-tubulin (both from Amersham) monoclonal antibody was added to each tube. The samples were incubated on ice for 1 h. The immunocomplexes were precipitated by adding protein G-Sepharose (Sigma) and the samples were incubated at 4°C with rocking for 1 h. The beads were collected by centrifugation and washed three times with lysis buffer. After the final wash, SDS sample buffer was added and the samples were proceeded for SDS-PAGE as described.

*Immunoblot analysis*

After electrophoresis, the gel was soaked in transfer buffer (50 mM Tris-borate, pH 8.3, 1 mM EDTA) for 10 min. Resolved proteins were then electro-transferred onto a nitrocellulose membrane (Hybond-C extra, Amersham) by a semi-dry method (OWL Scientific Plastics Inc.; Cambridge, MA). The membrane was incubated for 1 h with 3% gelatin in Tween containing Tris-buffered saline (TTBS: 20 mM Tris-HCl, pH 7.4, 500 mM NaCl, 0.05% Tween 20) and then rinsed with TTBS briefly. Subsequently, the membrane was incubated with monoclonal antibodies to HSP70 or β-tubulin (diluted 1:2,000 or 1:1,000 in TTBS containing 1% gelatin, respectively) at room temperature for 2 h. After three washes with TTBS, immunocomplexes on the membranes were reacted with goat anti-mouse antibody conjugated with alkaline phosphatase (diluted 1:2000 in TTBS containing 1% gelatin) at room temperature for 30 min. The membrane was then rinsed three times with TTBS, dried and developed into a colour immunoblot at room temperature in developing buffer (15 mg of nitro blue tetrazolium. 0.7% N,N-dimethylformamide, 30 mg of 5-bromo-4-chloro-3-indolyl phosphate per 100 ml, 1 mM $MgCl_2$, and 100 mM $NaHCO_3$, pH 9.8).

### Results

*Cytotoxicity of vincristine on cells pre-treated at supraoptimal temperature*

When the exponential growing 9L rat brain tumour cells were exposed to vincristine, the surviving fraction decreased as the drug concentration increased (Figure 1). However, if the cells were pre-treated at 45°C for 15 min and then recovered under normal growing conditions for 4, 8, and 12 h, the cell survivals were higher than those of the untreated controls. Cells recovered for 8 h were the most resistant to vincristine. At the challenge dose of $10^{-6}$ M vincristine for 1 h, the survivals of the cells recovered for 4, 8, 12 h were 35, 57, and 43%, respectively. The $ED_{50}$ for the cells recovered for 8 h was $3 \times 10^{-6}$ M, which was 100 times of the cells recovered for 4 h and 1,000 times of the untreated cells (Figure 1). Table I showed that the cell numbers remained relatively constant after different treatment protocols. The data clearly indicated that a large proportion of the pre-

**Figure 1** Resistance to vincristine of the heat-treated cells. Cells were pretreated at 45°C for 15 min and let recover at normal growing conditions for 4 (■), 8 (●) or 12 (○) h before they were challenged with different concentrations (as indicated) of vincristine for 1 h. Non-preheated cells (□) were included for comparison. Points and bars represent mean ± standard deviation from three independent experiments.

**Table I** Relative numbers of cells before and after vincristine treatments

| | *Before vincristine treatment* | *After vincristine treatment* $1 \times 10^{-5}$ M | $1 \times 10^{-6}$ M |
|---|---|---|---|
| Vincristine only | 1.00 | 1.02 ± 0.13 | 1.04 ± 0.07 |
| Recovery durations (h) after heat shock | | | |
| 4 | 1.00 ± 0.04 | 1.04 ± 0.05 | 1.03 ± 0.02 |
| 8 | 1.00 ± 0.03 | 1.11 ± 0.07 | 1.07 ± 0.05 |
| 12 | 1.11 ± 0.04 | 1.06 ± 0.04 | 1.10 ± 0.08 |

Cells were preheated at 45°C for 15 min and allowed to recover at normal growing conditions for various durations. Before or after vincristine challenge for 1 h, the cells were washed, trypsinised, and cell numbers were then counted. Values presented were the relative numbers of cells compared to that of non-heated controls. Means ± standard deviations were from three individual experiments.

heated cells became insensitive to vincristine were due to the pre-treatment of heat-shock and the subsequent recovery periods. In other words, the decrease in sensitivity to vincristine were not due to a selective killing of sensitive population which were lost during heat/drug treatments.

*Organisation of microtubules in the normal and pre-heated cells after vincristine treatment*

Cells heat-treated and recovered for 8 h were exposed to $10^{-6}$ M vincristine for 1 h. After treatments, the cells were processed for immunofluorescence microscopy using anti-$\beta$-tubulin as the primary antibody. Destruction of microtubules was clearly detected in non-heat-treated cells after they were exposed to vincristine (Figure 2a and b). In contrast, organisation of microtubules remained relatively stable in the vincristine-treated cells which were pre-treated at 45°C for 15 min and recovered at normal conditions for 8 h (Figure 2c and d).

*Levels of the induced HSPs and the cell survivals after vincristine treatment*

Analysis of protein patterns of the heat-treated cells during the recovery period showed that four HSPs, with molecular weights of 110, 90, 72 and 70 kDa were induced (Figure 3a). The induction kinetics and the levels of each HSP were different. Among the HSPs, the induction of HSP70 was highly responsive to the initial heat-treatment. Its maximal level of accumulation was reached at 8 h after the initial heat treatment and the accumulation level was 5-fold higher than that of the control cells (Figure 3b). In contrast, the induction of HSP72 was only slightly responsive. The optimal expression of this protein arrived after 4 h of recovery and the level was only 10% higher than that of the controls (Figure 3b). In addition, it was found that the basal expression of HSP90 and 110 was also higher than that of HSP70 and their induction were also less responsive. Their optimal levels of accumulation were only 2-fold compared to those of the control cells (Figure 3b).

The amount of each HSP during the recovery period as shown above was correlated to the surviving fractions of the drug- and heat-treated cells (data were calculated from Figure 1). It was found that only the level of HSP70 was well correlated to the survival data. The correlation coefficiency ($r^2$) between HSP70 and the surviving fractions varied from 0.91 to 0.95, depending on the dose of vincristine used (Figure 4a). On the other hand, the $r^2$ among the levels of HSP72, 90, as well as 110 and cell survivals were lower than 0.65 (Figure 4b to d), which were much lower than that of HSP70. The result indicated that the level of HSP70 was the best correlated to the induction of vincristine resistance generated by heat-shock treatment.

**Figure 2** Distribution of microtubules of normal and thermotolerant cells challenged with vincristine. Cells were pretreated at 45°C for 15 min and let recover at normal growing condition for 8 h before they were exposed to $10^{-6}$ M of vincristine for 1 h. After fixing, the samples were probed with anti-$\beta$-tubulin and processed for immunofluorescence microscopy. **a**, non-heated cells, **b**, vincristine-treated cells, **c**, pre-heated cells after 8 h of recovery, **d**, cells in **c**, challenged with vincristine. Magnification × 1,500.

**Figure 3** Protein patterns **a**, and the amount of HSPs **b**, of heat-treated cells. **a**, Cells were labelled with $^{35}$S-methionine for 20 h and then proceeded as described in Figure 1. At the end of the recovery periods, cells were lysed and the lysates were subjected to SDS-PAGE. Lane 1: Untreated cells (control). Lanes 2 to 5, cells were heat-treated and allowed to recover for 0, 4, 8, and 12 h, respectively. HSPs synthesised were indicated on the right. **b**, Relative amounts of HSPs were derived from the autoradiographs as shown in Figure 3a. HSPs: 70 (○), 72 (●), 90 (□) and 110 (■). Points and bars represent mean ± standard deviation from three independent experiments.

**Figure 4** Correlation of the amounts of HSPs and the surviving fractions of the pre-heated cells challenged with various concentrations of vincristine. The relative amounts of HSPs were obtained from Figure 3 and the survival data were from Figure 1. Vincristine concentrations: $10^{-5}$ (■), $10^{-6}$ (□), $2.5 \times 10^{-8}$ (●) and $2.5 \times 10^{-9}$ M (○). HSP70, $r^2 = 0.91$ to 0.94; HSP72, $r^2 =$ 0.;63 to 0.67; HSP90, $r^2 = 0.51$ to 0.64; HSP110, $r^2 = 0.30$ to 0.40.

**Figure 5** Co-precipitation of HSP70 and tubulins by anti-HSP70 monoclonal antibody. Cells were labelled with $^{35}$S-methionine for 20 h, treated at 45°C for 15 min and recovered at normal growing condition for various durations. Antibodies were added to the cell lysates and the immunocomplexes were precipitated with protein G-Sepharose. After washing, proteins remained on the beads were resolved by SDS-PAGE and the gels were processed for immunoblotting analysis using anti-$\beta$-tubulin as a probe. The membrane was then exposed to X-ray film and processed for autoradiography. Shown are the autoradiograph **a**, and the corresponding immunoblot **b**. Lane 1: Untreated cells (control). Lanes 2 to 5, cells were heat-treated and let recover for 0, 4, 8, and 12 h, respectively.

**Figure 6** Co-precipitation of HSP70 and tubulins by anti-$\beta$-tubulin monoclonal antibody. Samples were processed as described in Figure 5, but that anti-$\beta$-tubulin was used for immunoprecipitation and that anti-HSP70 was used as the primary antibody in the immunoblotting analysis. Shown are the autoradiograph **a**, and the corresponding immunoblot **b**. Lanes: the same as those in Figure 5.

*Association of HSP70 with tubulin in the heat-treated cells*

After heat treatment and recovery as described above, the cells were lysed and the cell lysates were immunoprecipitated with anti-HSP70 antibody. The immunoprecipitated proteins were resolved by SDS-PAGE, blotted onto a cellulose membrane and processed for immuno-analysis and autoradiography. In addition to HSP70, several proteins including tubulin were co-precipitated by the antibody (Figure 5a). The immunoprecipitation of tubulin in the presence of HSP70 by the anti-HSP70 antibody was further supported by immunoblotting analysis (Figure 5b). It was found that both HSP70 and tubulin were immunoprecipitated at highest level by anti-HSP70 antibody after 8 h of the initial heat-treatment. In reciprocal experiments, the cell lysates were immunoprecipitated with anti-$\beta$-tubulin antibody. Interestingly, both tubulin and HSP70 were immunoprecipitated by anti-$\beta$-tubulin. The highest level of HSP70 was also detected after 8 h of the heat-treatment (Figure 6). These data clearly indicated that HSP70 and tubulin were tightly associated during the recovery period.

### Discussion

We have demonstrated that cells treated at supraoptimal temperature synthesised HSP70 and developed vincristine resistance during the recovery period. HSP70 is the most prominent protein induced by a variety of stress conditions in all cells investigated (Schlesinger, 1990; Pelham, 1990). Although its physiological function is not yet fully understood, several lines of evidence suggested that synthesis of this protein is responsible for the development of thermotolerance of the cells (Li & Laszlo, 1985; Riabowol *et al.*, 1988; Johnston & Kucey, 1988). Most recently, it has been shown that cells transfected with *hsp*70 governed by a constitutive promoter were more resistant to heat-shock, directly supporting the above notion (Angelidis *et al.*, 1991; Li *et al.*, 1991). In the present studies, the 9L rat brain tumour cells were heated and recovered under normal growing conditions. During the course of recovery, the surviving cells synthesised HSP70, 72, 90 and 100. Simultaneously, the cells developed thermotolerance (unpublished data). It is interesting to note that the induction kinetics of HSP70 was coincident with the development of thermotolerance as well as vincristine resistance, i.e., cells at thermotolerant states are also resistant to vincristine.

The development of thermotolerance in cells after heat-shock treatment is an intriguing problem. It has been suggested that reorganisation and/or stabilisation of cytoskeleton after the heat-shock treatment is one of, if not the major reasons for development of thermotolerance (Wiegant et al., 1987). It has also been shown that external peptides which aid cell attachment and spreading were able to enhance thermotolerance of the cells (Sauk et al., 1990). These results indicated that integrity of cytoskeleton is a prerequisite for thermotolerance. Using vincristine as a tool, the integrity of microtubules before and after the initial heat-treatment were compared. Our results showed that microtubules in heat-treated cells are more resistant to the destruction by vincristine, further indicating that microtubules are more stable at the thermotolerant cells and that HSP70 may be involved in these processes. However, similar function has been proposed for HSP90. Like HSP72, HSP90 is constitutively expressed in unstressed cells. It can bind to specific polypeptides and either silence their function (e.g., glucocorticoid receptor) (Catelli et al., 1985; Sanchez et al., 1985), and/or escort them to their proper cellular compartment (e.g. $pp60^{src}$) (Oppermann et al., 1981; Brugge, 1986), a function similar to that of the members in the HSP70 family. HSP90 also binds to actin filaments (Nishida et al., 1986) and microtubules (Redmond et al., 1989), suggesting that it may be involved in the organisation/stabilisation of the cytoskeleton. Nevertheless, our data showed that among all HSPs induced, the level of HSP70 shows the best correlation with the survivals of the cells after they were challenged with vincristine. Here, vincristine resistance is considered to be an indicator for the integrity of microtubules. The mRNA for a 68-kDa microtubule-associated protein in rat brain was found to be hybridized with the *Drosophila* gene for the HSP70, implying that HSP70 may function as a microtubule-associated protein (Lim et al., 1984). More recently, it has been reported that HSP70 binds to actin and a number of proteins (Margulis & Welsh, 1991). The above observations, together with the present findings, strongly indicated that HSP70 may functinally associate with microtubules and other cytoskeletal components. Thus it may be responsible for the structural thermotolerance of the heat-treated cells.

By means of immunoprecipitation, it has been shown that HSP70 and tubulin are associated with each other. Together with the protective roles of HSP70 whenever it binds to other cellular proteins, it is conceivable that HSP70 also protects tubulin from being damaged by further stress. Although it has been suggested that HSP70 may be involved in the stabilisation of the cytoskeleton in its presence, it is the first time that its association with microtubules is directly demonstrated. HSP72 was also found to be able to associate with components of the cytoskeleton including the intermediate filaments and microtubules (Napolitano et al., 1985, 1987). Therefore, our data further support the notion that HSP70 and HSP72 are functionally similar (Lindquist & Craig, 1988). However, based on the facts that they differ in basal expression, the kinetics of induction after stresses, as well as the correlation with thermotolerance and vincristine resistance, their physiological functions may not be exactly the same. HSP70 and HSP72 may be functionally similar at the molecular level but they may have different physiological roles.

Drugs bound specifically to tubulin are useful in the therapy of specific diseases, e.g., vincristine is used individually or in combination with other drugs in the treatment of malignancies (Schiff & Horwitz, 1981; Ingle et al., 1989; Rarick et al., 1991). On the other hand, hyperthermia is also used in cancer therapy, either alone or in combination with other drugs (Hahn, 1982; Hornback, 1984; Anghileri & Rober, 1986). The current finding that hyperthermia could induce resistance to certain drugs (i.e. vincristine, and presumably other vinca alkaloids with similar functions), in addition to thermotolerance, indicating that hyperthermia and drug may cross-react. The finding is noteworthy in combining hyperthermia and drug treatment in the management of malignant diseases which warrants further investigations.

This work was supported by grants to Y.-K.L. from the R.O.C. National Science Council (NSC 81-0211-B-007-04 and 81-0418-B-007-01). The authors wish to thank Dr S.-D. Yang for helpful discussions.

### References

ANGELIDIS, C.E., LAZARIDIS, I. & PAGOULATOS, G.N. (1991). Constitutive expression of heat-shock protein 70 in mammalian cells confers thermoresistance. *Eur. J. Biochem.*, **199**, 35–39.

ANGHILERI, L.J. & ROBER, J. (1986). *Hyperthermia in Cancer Treatment*. CRC Press: Florida.

BECKMANN, R.P., MIZZEN, L.A. & WELCH, W.J. (1990). Interaction of Hsp 70 with newly synthesized proteins: implications for protein folding and assembly. *Science*, **248**, 850–854.

BLACK, A.R. & SUBJECK, J.R. (1990). Mechanism of stress-induced thermo- and chemotolerances. In *Stress Proteins*. Schlesinger, M.J., Santoro, M.G. & Garaci, E. (eds) pp.101–117. Springer-Verlag: Berlin.

BOWMAN, L.C., HOUGHTON, J.A. & HOUGHTON, P.J. (1986). GTP influences the binding of vincristine in human tumor cytosols. *Biochem. Biophys. Res. Commun.*, **135**, 695–700.

BRUGGE, J.S. (1986). Interaction of the Rous sarcoma virus protein, $pp60^{src}$, with the cellular proteins pp50 and pp90. *Curr. Topics Microbiol. Immunol.*, **123**, 1–23.

CATELLI, M.G., BINART, N., JUNG-TESTAS, I., RENOIR, J.M., BAULIEU, E.E., FERAMISCO, J.R. & WELCH, W.J. (1985). The common 90KD protein component of nontransformed '8S' steroid receptors is a heat shock protein. *EMBO J.*, **4**, 3131–3137.

CHAPPELL, T.G., WELCH, W.J., SCHLOSSMANN, D.M., PATER, K.B., SCHLESINGER, M.J. & ROTHMAN, J.E. (1986). Uncoating ATPase is a member of the 70 kilodalton family of stress proteins. *Cell*, **45**, 3–13.

CHIRICO, W.J., WATERS, M.G. & BLOBEL, G. (1988). 70 K heat shock-related proteins stimulate protein translocation into microsomes. *Nature*, **332**, 805–810.

COSS, R.A., DEWEY, W.C. & BAMBURG, J.R. (1982). Effects of hyperthermia on dividing Chinese hamster ovary cells and on microtubules *in vitro*. *Cancer Res.*, **42**, 1059–1071.

CREASEY, W.A. (1979). The vinca alkaloids. In *Antibiotics. Vol. 5*. Hahn, F.E. (ed.) pp.414–438. Springer-Verlag: New York.

DELUCA-FLAHERTY, C., MCKAY, D.B., PARHAM, P. & HILL, B.L. (1990). Uncoating protein (hsc70) binds a conformationally labile domain of clathrin light chain $LC_a$ to stimulate ATP hydrolysis. *Cell*, **62**, 875–887.

DESHAIES, R.J., KOCH, B.D., WERNER-WASHBURNE, M., CRAIG, E.A. & SCHEKMAN, R. (1988). A subfamily of stress proteins facilitates translocation of secretory and mitochondrial precursor polypeptides. *Nature*, **332**, 800–805.

DONOSO, J.A., HASKIN, K.M. & HIMES, R.H. (1979). Effect of microtubule-associated proteins on the interaction of vincristine with microtubules and tubulin. *Cancer Res.*, **39**, 1604–1610.

GUPTA, R.S. (1990). Microtubules, mitochondria, and molecular chaperones: a new hypothesis for *in vivo* assembly of microtubules. *Biochem. Cell Biol.*, **68**, 1352–1363.

HAHN, G.H. & LI, G.C. (1990). Thermotolerance, thermoresistance, and thermosensitization. In *Stress Proteins in Biology and Medicine*. Morimoto, R.I., Stissieres, A. & Georgopoulos, C. (eds) pp.79–100. Cold Spring Harbor Laboratory Press: New York.

HAHN, G.H. (1982). *Hyperthermia and Cancer*. Plenum Press: New York.

HIGHTOWER, L.E. & WHITE, F.P. (1981). Cellular responses to stress: comparison of a family of 71–73-kilodalton proteins rapidly synthesized in rat tissue slices and canavanine-treated cells in culture. *J. Cell Physiol.*, **108**, 261–275.

HORNBACK, N.B. (1984). *Hyperthermia and Cancer: Human Clinical Trial Experience*. CRC Press: Florida.

INGLE, J.N., MAILLIARD, J.A., SCHAID, D.J., KROOK, J.E., GERSTNER, J.B., PFEIFLE, D.M., MARSCHKE, R.G. Jr, LONG, H.J., MCCORMACK, G.W. & FOLEY, J.F. (1989). Randomized trial of doxorubicin alone or combined with vincristine and mitomycin C in women with metastatic breast cancer. *Am. J. Clin. Oncol.*, **12**, 474–480.

JOHNSTON, R.N. & KUCEY, B.L. (1988). Competitive inhibition of hsp70 gene expression causes thermosensitivity. *Science*, **242**, 1551–1554.

LAEMMLI, U.K. (1970). Cleavage of structural proteins during the assembly of the head of bacteriophage T4. *Nature*, **227**, 680–685.

LEE, W.-C., LIN, K.-Y., CHEN, C.-M., CHEN, Z.-T., LIU, H.J. & LAI, Y.-K. (1991). Induction of heat-shock response and alterations of protein phosphorylation by a novel topoisomerase II inhibitor, withangulatin A, in 9L rat brain tumor cells. *J. Cell Physiol.*, **149**, 66–76.

LI, G.C., LI, L., LIU, Y.-K., MAK, J.Y., CHEN, L. & LEE, W.M.F. (1991). Thermal response of rat fibroblasts stably transfected with the human 70-kDa heat shock protein-encoding gene. *Proc. Natl Acad. Sci. USA*, **88**, 1681–1685.

LI, G.C. & LASZLO, A. (1985). Amino acid analogs while inducing heat shock proteins sensitize CHO cells to thermal damage. *J. Cell Physiol.*, **122**, 91–97.

LI, G.C. & MAK, J.Y. (1985). Induction of heat shock protein synthesis in murine tumors during the development of thermotolerance. *Cancer Res.*, **45**, 3816–3824.

LIM, L., HALL, C., LEUNG, T. & WHATLEY, S. (1984). The relationship of the rat brain 68 kDa microtubule-associated protein with synaptosomal plasma membranes and with the *Drosophila* 70kDa heat-shock protein. *Biochem. J.*, **224**, 677–680.

LIN, R.S., TURI, A., KWOCK, L. & LU, R.C. (1982). Hyperthermic effect on microtubule organization. *Natl Cancer Inst. Monogr.*, **61**, 57–61.

LINDQUIST, S. & CRAIG, E.A. (1988). The heat-shock proteins. *Annu. Rev. Genet.*, **22**, 631–677.

MARGULIS, B.A. & WELSH, M. (1991). Analysis of protein binding to heat shock protein 70 in pancreatic islet cells exposed to elevated temperatures or interleukin 1$\beta$. *J. Biol. Chem.*, **266**, 9295–9298.

MORIMOSTO, R.I., TISSIERES, A. & GEORGOPOULOS, C. (1990). *Stress Proteins in Biology and Medicine*. Cold Spring Harbor Laboratory Press: New York.

NAPOLITANO, E.W., PACHTER, J.S., CHIN, S.S.M. & LIEM, R.K.H. (1985). $\beta$-internexin, a ubiquitous intermediate filament-associated protein. *J. Cell Biol.*, **101**, 1323–1331.

NAPOLITANO, E.W., PACHTER, J.S. & LIEM, R.K.H. (1987). Intracellular distribution of mammalian stress proteins. *J. Biol. Chem.*, **262**, 1493–1504.

NISHIDA, E., KOYASU, S., SAKAI, H. & YAHARA, I. (1986). Calmodulin-regulated binding of the 90-kDa heat shock protein to actin filaments. *J. Biol. Chem.*, **261**, 16033–16036.

NOBLE, R.L. (1990). The discovery of the vinca alkaloids-chemotherapeutic agents against cancer. *Biochem. Cell Biol.*, **68**, 1344–1351.

OPPERMANN, H., LEVINSON, W. & BISHOP, J.M. (1981). A cellular protein that associates with a transforming protein of Rous sarcoma virus is also a heat shock protein. *Proc. Natl Acad. Sci. USA*, **78**, 1067–1071.

PELHAM, H.R.B. (1986). Speculations on the functions of the major heat shock and glucose-regulated proteins. *Cell*, **46**, 959–961.

PELHAM, H.R.B. (1990). Functions of the hsp70 protein family: an overview. In *Stress Proteins in Biology and Medicine*. Morimoto, R.I., Tissieres, A. & Georgopoulos, C. (eds) pp.287–299. Cold Spring Harbor Laboratory Press: New York.

RARICK, M.U., GILL, P.S., MONTGOMERY, T., BERNSTEIN-SINGER, M., JONES, B. & LEVINE, A.M. (1991). Treatment of epidermic Kaposi's sarcoma with combination chemotherapy (vincristine and bleomycin) and zidovudine. *Ann. Oncol.*, **1**, 147–149.

REDMOND, T., SANCHEZ, E.R., BRESNICK, E.H., SCHLESINGER, M.J., TOFT, D.O., PRATT, W.B. & WELSH, M. (1989). Immunofluorescence colocalization of the 90-kDa heat-shock protein and microtubules in interphase and mitotic mammalian cells. *Eur. J. Cell Biol.*, **50**, 66–75.

RIABOWOL, K.T., MIZZEN, L.A. & WELCH, W.J. (1988). Heat shock is lethal to fibroblasts microinjected with antibodies against hsp70. *Science*, **242**, 433–436.

SANCHEZ, E.R., TOFT, D.O., SCHLESINGER, M.J. & PRATT, W.B. (1985). Evidence that the 90 kDa phosphoprotein associated with the untransformed L-cell glucocorticoid receptor is a murine heat shock protein. *J. Biol. Chem.*, **260**, 12358–12403.

SAUK, J.J., VAN KAMPEN, C.L., NORRIS, K., MOEHRING, J., FOSTER, R.A. & SOMERMAN, M.J. (1990). Persistent spreading of ligament cells on osteopontin/bone sialoprotein-I or collagen enhances tolerance to heat shock. *Exp. Cell Res.*, **188**, 105–110.

SCHIFF, P.B. & HOROWITZ, S.B. (1981). Tubulin: a target for chemotherapeutic agents. In *Molecular Actions and Target for Cancer Chemotherapeutic Agents*, Sartorelli, A.C., Lazo, J.S. & Bertino, J.R. (eds) pp.483–507. Academic Press: New York.

SCHLESINGER, M.J. (1990). Heat shock proteins. *J. Biol. Chem.*, **265**, 12111–12114.

SCHLESINGER, M.J., SANTORO, M.G. & GARACI, E. (1990). *Stress Proteins*. Springer-Verlag: Berlin.

TAYLOR, W.I. & FARNSWORTH, N.R. (1975). *The Catharanthus Alkaloids*. Dekker: New York.

WEIZSAECKER, M., DEEN, D.F., ROSENBLUM, M.L., HOSHINO, T., GUTIN, P.H. & BARKER, M. (1981). The 9L rat brain tumor: description and application of an animal model. *J. Neurol.*, **224**, 183–192.

WELCH, W.J. & SUHAN, J.P. (1985). Morphological study of the mammalian stress response: characterization changes in cytoplasmic organelles, cytoskeleton and nucleoli and appearance of intranuclear actin filaments in rat fibroblasts after heat shock treatment. *J. Cell Biol.*, **101**, 1198–1211.

WIEGANT, F.A.C., VAN BERGEN EN HENEGOUWEN, P.M., VAN DONGEN, G. & LINNEMANS, W.A.M. (1987). Stress-induced thermotolerance of the cytoskeleton of mouse neuroblastoma cells and rat Reuber H35 hepatoma cells. *Cancer Res.*, **47**, 1674–1680.